# Hearing Loss and Risk of Dementia: A Burden of Proof Study

**DOI:** 10.1002/alz70860_101600

**Published:** 2025-12-23

**Authors:** Anh Thy Nguyen, Natalie Chen, Channa Buxbaum, Kate Gillespie, Liane Ong, Jamie Steinmetz

**Affiliations:** ^1^ Institute for Health Metrics and Evaluation, Seattle, WA, USA

## Abstract

**Background:**

Hearing loss is a significant public health concern, which we estimate in the Global Burden of Disease (GBD) study impacts over one billion or 65% of adults aged 55 and older. The Lancet Commission identified a link between hearing loss and increased risk of dementia, however, prior evidence synthesis have not considered non‐linear relationships nor have they incorporated between‐study variation into uncertainty estimates. We conducted a systematic review and meta‐analysis to quantify the relative risk of dementia associated with hearing loss, utilizing a novel meta‐analytic framework to address these prior limitations.

**Method:**

We searched PubMed, Embase, and Web of Science for articles that estimated all‐cause dementia risk associated with adult hearing loss from January 1980 to October 2023. To summarize the evidence, we used the Burden of Proof (BoP) meta‐analytic framework developed for the GBD study. Unlike traditional meta‐analysis, the BoP methodology adjusts for systematic biases, allows for non‐linear fit, and incorporates between‐study variation in uncertainty estimates to provide a conservative interpretation of the evidence. We estimated dementia risks along continuous decibel thresholds in a dose‐response model among studies that use audiometry. Mean relative‐risks (RR) and 95% uncertainty intervals (UI) accounting for between‐study heterogeneity are presented.

**Result:**

We screened 808 records and identified 38 studies for inclusion, covering 481,809 dementia cases. Hearing loss was measured by self‐report in 9 studies, administrative records in 12 studies, audiometry in 10 studies, and other methods of direct observation in 7 studies. Among studies that use audiometry, compared to a baseline threshold of 15 decibel, dementia risk is increased by 29% (95% UI: 23‐35%) for moderate hearing loss (35 decibel threshold) and 49% (95% UI: 39 – 61%) for severe hearing loss (65 decibel threshold). We also defined hearing loss as a binary variable to incorporate alternative hearing loss measurements and results yielded a non‐significant increase in risk of dementia: 31% (95% UI: ‐4 – 80%).

**Conclusion:**

Hearing loss in adults is associated with increased risk for dementia, with stronger evidence among studies that use objective audiometry measurements. Dementia risk reduction should prioritize preventing hearing loss and promoting hearing aid use and access.

